# SPARSE: Seed Point Auto‐Generation for Random Walks Segmentation Enhancement in medical inhomogeneous targets delineation of morphological MR and CT images

**DOI:** 10.1120/jacmp.v16i2.5324

**Published:** 2015-03-08

**Authors:** Haibin Chen, Xin Zhen, Xuejun Gu, Hao Yan, Laura Cervino, Yang Xiao, Linghong Zhou

**Affiliations:** ^1^ Department of Biomedical Engineering Southern Medical University Guangzhou 510515 China; ^2^ Department of Radiation Oncology The University of Texas, Southwestern Medical Center Dallas TX USA; ^3^ Center for Advanced Radiotherapy Technologies and Department of Radiation Medicine and Applied Sciences University of California San Diego La Jolla CA USA

**Keywords:** segmentation, inhomogeneous target, random walks, seed point, autogeneration

## Abstract

In medical image processing, robust segmentation of inhomogeneous targets is a challenging problem. Because of the complexity and diversity in medical images, the commonly used semiautomatic segmentation algorithms usually fail in the segmentation of inhomogeneous objects. In this study, we propose a novel algorithm imbedded with a seed point autogeneration for random walks segmentation enhancement, namely SPARSE, for better segmentation of inhomogeneous objects. With a few user‐labeled points, SPARSE is able to generate extended seed points by estimating the probability of each voxel with respect to the labels. The random walks algorithm is then applied upon the extended seed points to achieve improved segmentation result. SPARSE is implemented under the compute unified device architecture (CUDA) programming environment on graphic processing unit (GPU) hardware platform. Quantitative evaluations are performed using clinical homogeneous and inhomogeneous cases. It is found that the SPARSE can greatly decrease the sensitiveness to initial seed points in terms of location and quantity, as well as the freedom of selecting parameters in edge weighting function. The evaluation results of SPARSE also demonstrate substantial improvements in accuracy and robustness to inhomogeneous target segmentation over the original random walks algorithm.

PACS number: 87.57.nm

## I. INTRODUCTION

Segmentation of medical images is a significant and challenging task for disease diagnosis^1^, ^2^ and treatment planning.^3^, ^4^, ^5^ It can be roughly classified into three categories, namely, manual, semiautomatic (interactive), and automatic segmentation.^(6)^ Manual segmentation is usually time‐consuming and experience dependent. Automatic segmentation cannot guarantee optimal results due to the complexity and diversity of the medical images, and often requires a physician's further intervention. Interactive segmentation,^(7)^ which allows the physician to incorporate their professional knowledge and the specific clinical criteria and saves physicians' time with computer aid, is more attractive than other two.

In the past decade, efforts have continued towards developing interactive segmentation methods. One of the popular interactive segmentation methods is random walks (RW),^(8)^ where the users achieve segmentation through estimating the probability that a random walker starting at each voxel to reach the labeled initial seed points (SPs). The RW algorithm has several drawbacks in practice. Firstly, RW relies on the location and quantity of the initial SPs to a large extent. It has been demonstrated that RW is stable to changes of the SPs only when the changes of location and quantity are below 10% and 50%, respectively.^(9)^ Moreover, to obtain satisfactory segmentation results, the quantity of initial SPs should be sufficient to be representative of almost all intensity levels in the target. However, in practice, it is tedious and laborious to place sufficient SPs on the target, especially in a 3D scenario. In this sense, a stable segmentation result may not be guaranteed when insufficient SPs are used, especially for medical inhomogeneous targets, which are common in clinical studies.

Great efforts have been made to improve RW by reducing its reliance on the SPs. Dong et al.^(10)^ proposed a novel SPs selection method composed of a region growing technique and morphological operation to promote the RW segmentation of ventricle in 3D dataset. But, it is limited to cavity or cavity wall extraction. Li et al.^(11)^ extended the RW by incorporating a prior shape to relieve the requirement of the SPs. However, this method works under the assumptions of slight occlusion, similar background, and illumination changes in the pedestrian images, which has limited application in medical images. Moreover, a proper prior shape is usually difficult to obtain because of the diversified shapes of clinic targets. Onoma et al.^(12)^ proposed an improved RW by initializing the SPs automatically via fuzzy‐C means to yield better segmentation of lung tumor. However, it fails in the segmentation of lesions with complex shapes and inhomogeneous uptake. Cui et al.^(13)^ described a SPs automatic selection strategy for RW‐based segmentation of lung tumor in computed tomography (CT) image by using the positron emission tomography (PET) image as the prior. But the availability of the PET image may restrain its application to segmentation tasks in other image modalities.

In this paper, we propose a novel algorithm imbedded with seed point autogeneration for random walks segmentation enhancement (named SPARSE). A previous study^(9)^ has shown that more SPs generally provide more useful information in the target region and background, and thus more robust segmentation results can be guaranteed. With a limited number of user‐labeled initial SPs, we have developed a SPs autogeneration scheme to obtain extended seed points (ESPs) by estimating the probability of each voxel with respect to a certain label. The RW algorithm is then applied upon the ESPs to achieve improved segmentation result. The SPARSE is implemented using compute unified device architecture (CUDA) on a graphic processing unit (GPU) platform to improve computation efficiency. The performance of SPARSE is evaluated using homogeneous and inhomogeneous cases. It is found that the SPARSE is robust to the initial SPs in terms of location, quantity, and the selection of parameter *β* in edge weighting function. Furthermore, SPARSE improves the segmentation accuracy when compared with the original RW and another state‐of‐the‐art interactive segmentation algorithm — the graph cut (GC) method.^(14)^

## II. MATERIALS AND METHODS

### A. Review of the RW

RW ^8^, ^15^ is used to solve the segmentation problem by calculating the probability that a random walker starting at each voxel will first reach one of the labeled SPs. A graph consisting of a pair G=(V,E) with vertices (nodes) v∈V and edges e∈E is first created based on the image *I* to be segmented. The edge *e* spanning two vertices, $v_{i}$ and *v_j_,* is denoted by $e_{\text{ij}}$ . The connectivity of two adjacent vertices on an edge is weighted by *ω_ij_*, such as

(1) $$\omega_{ij}=\text{exp}\left(-\beta {\left(I_{i}-I_{j}\right)}^{2}\right)$$

where $I_{i}$ and $I_{j}$ indicate the image intensity at voxel *i, j,* respectively. *β* represents the only free parameter.

The desired random walker probabilities *x* on vertices v∈V can be obtained by solving the following combinatorial Dirichlet problem:

(2) $$D[x]=\frac{1}{2}x^{T}Lx=\frac{1}{2}\sum_{e_{ij}\in E}\omega_{ij}(x_{i}-{x_{j})}^{2}$$

where $x_{i}$ and $x_{j}$ are the random walker probabilities on vertices $v_{i}$, $v_{j}$, respectively. *L* is the Laplacian matrix:

(3) $$L_{ij}=\{\begin{array}{ll} d_{i} & if\;i=j,\\ -\omega_{ij} & \;if\;v_{i}\;and\\ 0 & otherwise. \end{array}\;v_{j}\;are\;adjacent\;vertices,$$

$d_{i}=\sum \omega_{ij}$ is the degree of a vertex for all edges $e_{\text{ij}}$ incident on $v_{i}$. The Laplacian matrix is a sparse matrix. It is built according to four‐connectivity and six‐connectivity for 2D and 3D scenarios, respectively.

The vertices can be partitioned into two sets, $V_{M}$ (labeled vertices) and $V_{U}$ (unlabeled vertices) such that $V_{M}\cup V_{U}=V$ and $V_{M}\cap V_{U}=\emptyset$. In this way, Eq. (2) can be decomposed into

(4) $$D[x_{U}]=\frac{1}{2}[x_{M}^{T}x_{U}^{T}]\left[\begin{array}{ll} L_{M} & B\\ B^{T} & L_{U} \end{array}\right]\left[\begin{array}{l} x_{M}\\ x_{U} \end{array}\right]$$

where $x_{M}$ and $x_{U}$ correspond to the probabilities of the labeled and unlabeled voxels, respectively. Differentiating $D[x_{U}]$ with respect to $x_{U}$ and finding the critical point yields

(5) $$L_{U}x_{U}=-B^{T}x_{M}$$

The probabilities $x_{U}$ for those unlabeled voxels can be easily calculated by solving the above sparse, positive definite linear Eq. (5). Denoting the probability assumed at node $v_{i}$ for each label *s,* by $x_{i}^{s}$, the final segmentation is obtained by assigning each node $v_{i}$ the label corresponding to maximum probability max_*s*_($x_{i}^{s}$). Usually, at least two groups of labels (s≥2) need to be supplied manually (i.e., SPs inside the region to be segmented (named foreground points) and SPs at the background (named background points)).

### B. Seed point autogeneration

In the 2D homogenous scenario, only a small number of SPs are needed to be labeled to yield a satisfactory segmentation. For the 3D segmentation task, it would be tedious and sometimes impractical to specify all the SPs on each slice of a 3D volume. Moreover, if the target to be segmented is not homogeneous, but contains highly diversified materials instead, using only a small number of SPs usually fails to provide enough information of the target, and thus satisfactory result cannot be obtained. In this study, we propose a SPs autogeneration scheme to obtain the ESPs for RW by estimating the probability of each voxel with respect to a certain label.

Specifically, let us assume that there are k(k≥2) labels $s_{k}$, and denote the labeled SPs and the corresponding intensities as $S^{s}k,\;t_{c}^{s}k$ respectively, where *c* is the number of the labeled SPs $S^{s}k$. The intensity distribution probability $p(i|s_{k})$ of voxel *i* in image *I* with respect to label is thus given by

(6) $$p(i|s_{k})=\frac{1}{N^{s_{k}}}{\sum_{c}e}^{-\frac{{\left(I_{i}-t_{c}^{s_{k}}\right)}^{2}}{\sigma }}$$

where *σ* is a tuning parameter that refects the rigor level of the similarity criteria, and $N^{s_{k}}$ is a normalizing constant for label $s_{k}$:

(7) $$N^{s_{k}}=\sum_{l=0}^{m}\sum_{c}e^{-\frac{{\left(l-t_{c}^{s_{k}}\right)}^{2}}{\sigma }}$$

where $m=\text{max}\;\text{max}(I_{i}-I_{j}),\forall i,j$.

Once $p(i|s_{k})$ is available, the probability $p(s_{k}|i)$ of voxel *i* to the chain of label $s_{k}$ can be simply calculated as

(8) $$p(s_{k}|i)=p(i|s_{k})/\sum_{s=s_{1}}^{s_{k}}p(i|s)$$

with 1 representing the highest probability. In this way, we can obtain the probability maps $P^{s_{k}}$ with respect to label $s_{k}$ for each voxel in image *I*.

**Figure 1:**
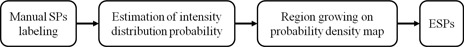
The flowchart of ESPs generation.

Region growing^(16)^ is then performed on the probability maps $P^{s_{k}}$ with the initially labeled SPs $S^{s}k$ as the SPs and $P(s_{k}|i)>P_{T}^{s_{k}}$ as the growing condition to yield the ESPs $E^{s_{k}}$, where $P_{T}^{s_{k}}$ is the threshold to filter those points with high similarity to the label $s_{k}$. For simplicity, we set $P_{T}^{s_{k}}=P_{T}$ for $\forall s_{k}$ in this study. The final segmentation is then completed by performing the RW with $E^{s_{k}}$ for each label $s_{k}$. The above ESPs generation scheme is summarized in Fig. 1.

### C. Implementation

The proposed algorithm is implemented on a platform equipped with an NVIDIA Tesla C1060 card with a total number of 240 1.3 GHz processors, and a 4 GB DDR3 memory shared by all processors. This platform enables parallel processing of the same operations on different CUDA threads simultaneously, which speeds up the entire algorithm. In order to efficiently parallelize RW in the CUDA environment, the data parallel portions of the algorithm are identified and grouped into the following kernels: 1) an edge kernel to build a graph with vertices and edges; 2) a weighting kernel to compute $\omega_{ij}$; 3) a normalization kernel to normalize $\omega_{ij}$; and 4) a Laplacian kernel to create the Laplacian sparse matrix. Moreover, we adopt the CUSP (a CUDA‐based library for sparse linear algebra and graph computations)^(17)^ to solve the sparse linear Eq. (6). Sparse matrices are stored using the Coordinate (COO) format. The conjugate gradient (CG) method is used as the iterative solver with relative tolerance of 10^‐6^ and maximum iteration 1000 as the stopping criteria.

### D. Evaluation data

**Figure 2:**
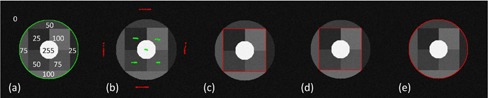
The synthetic phantom segmentation results. (a) A synthetic phantom with an inhomogeneous ground truth target (green contour). The overlaid digits indicate the intensities in each piecewise block. (b) Initial foreground (green) and background (red) SPs; (c) segmentation by RW; (d) segmentation by GC; (e) segmentation by SPARSE. The segmentations in (c) ~ (e) are shown in red contour.

The performance of SPARSE is evaluated using a synthetic phantom and four clinical 2D (cases 1‐4) and twenty (cases 5‐24) 3D CT/ MR images. The synthetic phantom (Fig. 2) contains a ground truth target which is made up of piecewise blocks with different intensity values representing inhomogeneous anatomical structures. The image resolution of the phantom is 256×256, and Gaussian noise with variance of 20 is added to the phantom, yielding a signal‐to‐noise ratio of roughly 25 in it. For clinical cases, Cases 1‐4 are 2D cases including: a fibula CT image of resolution 128×128 (case 1); a lung CT image of resolution 512×512 (case 2); a corpus callosum T2 MR image of resolution 320×320 (case 3); and an abdomen CT image of resolution 512×512 (case 4). Cases 5‐24 are 3D cases including: three high‐dose‐rate (HDR) brachytherapy CT images with tandem and cylinder (T&C) applicator of resolution 256×256×85 (cases 5, 11), 256×256×60 (case 12); five HDR CT images with tandem and ovoid (T&O) applicator of resolution 256×256×55 (case 6), 256×256×75 (case 13), 256×256×60 (case 14), 256×256×58 (case 15), and 256×256×82 (case 16); three T1c MR images of resolution 256×256×100 (cases 7, 17‐18); three T2, T1c, and FLAIR MR image series of resolution 160×216×176 (cases 8‐10, 19‐24). In this study, we classify all the evaluated cases into two categories, namely “homogeneous” and “inhomogeneous”, according to the variance of the intensity levels of the segmentation target. Specifically, given the ground truth segmentation, the intensity of the target is first normalized between [0,1], and the standard deviation (SD) is calculated. For those cases with target SD values smaller than a predefined threshold (0.1 is used in this study), they can be regarded as “homogeneous”; otherwise, they are classified as “inhomogeneous”. The target SD value for the synthetic phantom is around 0.22, and the target SD values for other clinical cases are listed in Table 1. By using this metric, we classify cases 3, 7 and 17‐18 as the homogeneous cases, while the others cases, including the synthetic phantom, as the inhomogeneous cases.

**Table 1:** The target SD values of all clinical cases

*Case #*	*SD*											
1‐12	0.17(+)	0.12(+)	0.08(−)	0.15(+)	0.21(+)	0.26(+)	0.09(−)	0.17(+)	0.18(+)	0.11(+)	0.16(+)	0.17(+)
13‐24	0.21(+)	0.19(+)	0.21(+)	0.19(+)	0.09(−)	0.09(−)	0.10(+)	0.12(+)	0.10(+)	0.11(+)	0.11(+)	0.10(+)

−=homogeneous; +=inhomogeneous

### E. Quantifications of segmentation performance

To quantitatively evaluate the segmentation performance, we employ three similarity metrics: the Dice's coefficient (DC),^18^, ^19^ the percent error (PE), and the Hausdorff distance (HD).^(20)^ Given the ground truth segmentation region *A* and the calculated segmentation region *B,* and their corresponding boundary point sets $\vec{A}=\{a_{1},\ldots ,a_{p}\}$ and $\vec{B}=\{b_{1},\ldots ,b_{q}\}$, the DC is defined as DC=2(A∩B)/(A+B), which ranges from 0 to 1, corresponding to the worst and the best segmentation, respectively. The PE is defined as PE=(A∪B−A∩B)/A with 0 representing the best segmentation. The HD is defined as $HD=\text{max}(h(\vec{A},\vec{B}),h(\vec{B},\vec{A})$, where $h(\vec{A},\vec{B})=\underset{a\in \vec{A}}{\text{max}}\underset{b\in \vec{B}}{\text{max}}\left\|a-b\right\|$ and ‖⋅‖ is the $L_{2}$ norm on the points of ‖⋅‖ and $\vec{B}$. For all the evaluated cases (expect for cases 7‐10 and cases 17‐24 where the ground truths are available), the targets are delineated manually by three experienced physicians, and the optimal combination of these three raters is estimated using the SPAPLE algorithm^(21)^ and serves as the surrogate of the ground truth segmentation for each case. All the generated ground truth segmentations are then used for segmentation accuracy studies between the RW, GC, and SPARSE with the above quantitative metrics.

## III. RESULTS

### A. Synthetic phantom results

Figure 2 illustrates the comparison results of the synthetic phantom using the RW, GC, and SPARSE algorithm. This simple phantom contains an inhomogeneous target made up of nine blocks with different intensities ranging from 25 to 255 (Fig. 2(a)), and the same SPs for RW, GC, and SPARSE are only seeded in five of them (Fig. 2(b)). We can see that the RW and GC algorithm both fail in segmenting the entire target blocks (Fig. 2(c) and (d)). The SPARSE, in contrast, yields a successful segmentation (Fig. 2(e)).

### B. Influence of initial seed points: quantity and location

**Figure 3:**
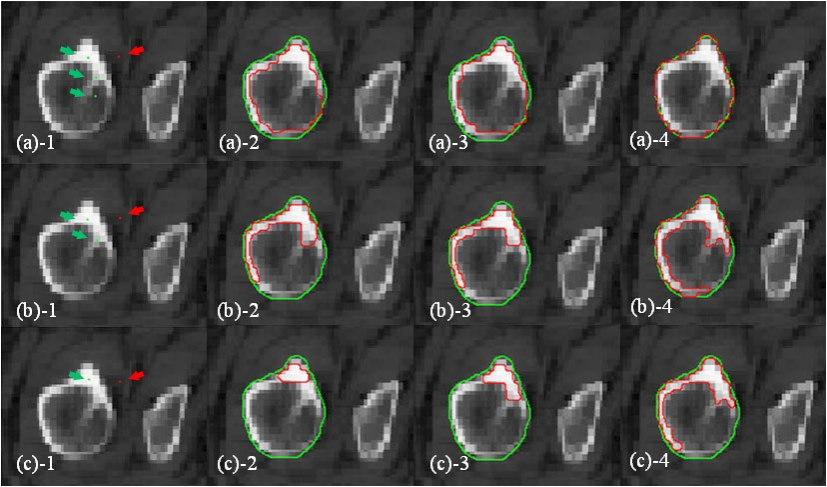
Fibula segmentation with different SPs (case 1). Segmentation of the fibula with one background SP outside of the fibula (red point), and three/two/one foreground SPs (green points in (a)−1, (b)−1, and (c)−1, respectively) inside the fibula. The red curves in (a)−2, (b)−2, and (c)−2, in (a)−3, (b)−3, and (c)−3, and in (a)−4, (b)−4, and (c)−4 represent the segmentation results using RW, GC, and SPARSE, respectively. The green curves are the ground truth segmentation.

**Figure 4:**
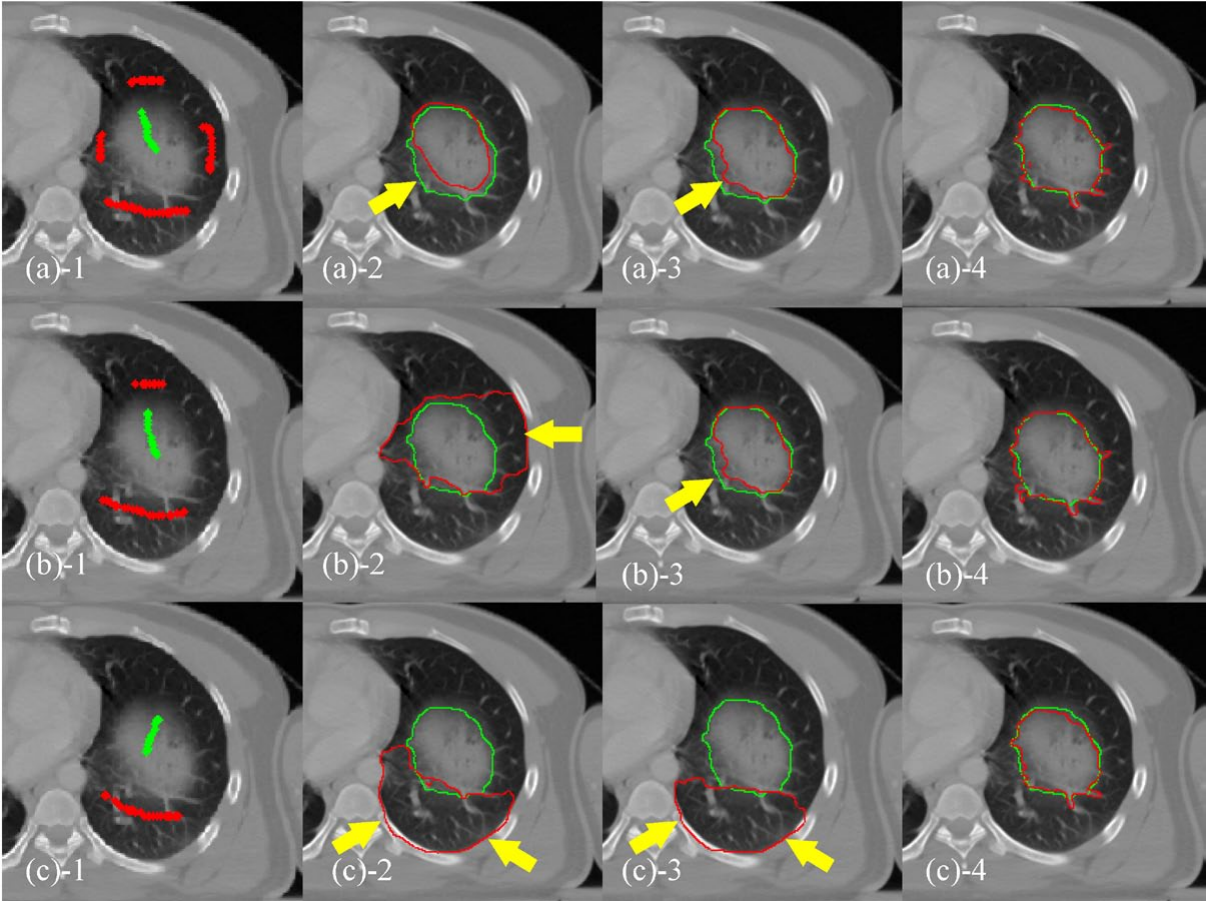
Lung tumor segmentation with different SPs (case 2). Segmentation of a lung tumor with sufficient ((a)−1), limited ((b)−1) and insufficient ((c)−1) foreground (green) and background (red) SPs. The red curves in (a) to (c)−2, (a) to (c)−3, and (a) to (c)−4 represent the segmentation results using RW, GC, and SPARSE, respectively. The green curves are the ground truth segmentation. The yellow arrows indicate the undersegmentation ((a)−2,−3, (b)−3), oversegmentation ((b)−2), and failed segmentation ((c)−2,−3).

Figure 3 demonstrates an example segmentation of the fibula using RW, GC, and SPARSE with different seed points (case 1). Figure 3(a) shows that RW, GC, and SPARSE can yield satisfactory segmentation results with sufficient SPs (Fig. 3(a) −2,−3,−4). When one (Fig. 3(b) −1) or two SPs (Fig. 3(c) −1) inside the fibula are removed, keeping the others at their original positions, RW, GC, and SPARSE deteriorate, although SPARSE behaves much better than RW and GC. Figure 4 shows another comparison of segmenting a lung tumor with SPs of different locations and quantities (case 2). Figure 4(a) shows that RW, GC, and SPARSE can yield similar satisfactory results when sufficient SPs are labeled (Fig. 4(a) −2,−3,−4). However, when reducing the number of the SPs as well as changing their locations (Figs. 4(b) −1, 4(c)−1), the segmentation deteriorates (Fig. 4(b) −2,−3), or even fails (Fig. 4(c) −2,−3) by RW and GC. In contrast, SPARSE produces more robust segmentation results (Figs. 4(a) −4 to (c)−4).

**Figure 5:**
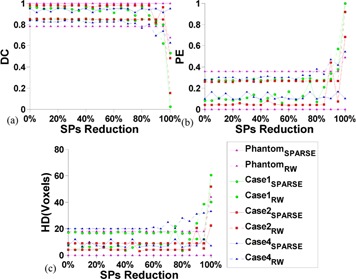
Sensitivity analysis of SPs' quantity using the synthetic phantom and cases 1, 2 and 4. DC (a), PE (b), and HD (c) are comparisons of the segmentation response to SPs reduction between RW and SPARSE, respectively. The x‐axis indicates the percentage of SPs reduction (5% interval) with respect to the initial seeding. Note that the 100% value corresponds to the scenario when only one SP is left.

The synthetic phantom and three challenging cases (cases 1, 2, and 4) with inhomogeneous targets are used for further assessment of the impact of SPs' quantity and location on segmentation performance. To analyze the sensitivity of SPs' quantity, certain amounts of initial SPs are first labeled on the images, and then gradually reduced by random down‐sampling with interval of 5% of the original number. The corresponding segmentation results are tracked on each reduction step and quantitatively measured by three similarity metrics: DC, PE, and HD. Figure 5 shows the comparison results of the segmentation response to SPs reduction, with RW and SPARSE. For all the evaluated cases, SPs reduction of less than 80% are seen to produce only minor changes in the resulting segmentation using both RW and SPARSE, while sharp drops in segmentation quality occur when the reduction is larger than 80%. Perturbations of segmentation performance in SPARSE is slightly larger than RW; however, SPARSE always has superior segmentation results than RW for all three metrics.

**Figure 6:**
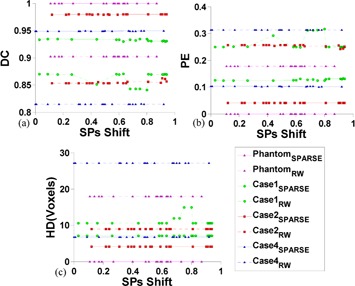
Sensitivity analysis of SPs' location using the synthetic phantom and cases 1, 2 and 4. DC (a), PE (b), and HD (c) are comparisons of the segmentation response to random SPs location between RW and SPARSE, respectively. Given certain initial labeled SPs, the shift amplitude of each SPs is given by multiplying the minimum distance from its current location to the boundary points on the ground truth segmentation with a random number ranges in (0,1), which is the x‐axis. The shift direction is randomly assigned.

To analyze the sensitivity of SPs' location, arbitrary SPs placement is simulated by shifting the labeled SPs with a random direction and amplitude. Specifically, given certain initial labeled SPs, the shift amplitude of each SPs is given by multiplying the minimum distance from its current location to the boundary points on the ground truth segmentation with a random number in the range (0,1). The shift direction is randomly assigned. Perturbing in this way, the initial SPs can only move within a reasonable distance without crossing over (e.g., moving the foreground points into the background). The segmentation results are tracked on each random SPs location and quantitatively measured by DC, PE, and HD. Figure 6 shows the comparison of segmentation performance relative to the SPs position changes between RW and SPARSE. In RW, a small variation of segmentation performance is observed in the synthetic phantom and in cases 1 and 2, but large perturbation is seen in case 4. In contrast, segmentation is stable for all the evaluated cases via SPARSE. Moreover, SPARSE behaves much better than RW in terms of segmentation accuracy.

### C. Influence of parameter β

**Figure 7:**
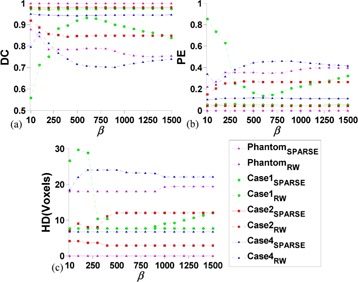
Sensitivity analysis of *β* using the synthetic phantom and case 1, 2 and 4. DC (a), PE (b), and HD (c) are comparisons of the segmentation response to different *β* (with same SPs) between RW and SPARSE, respectively. *β* increases from 10 to 1500 with interval size of 100.

Since the number of SPs is largely increased via SPs autogeneration, the influence of *β* is weakened in SPARSE. Figure 7 shows experiments of the segmentation response to different *β*. It is shown that the segmentation results vary greatly when different *β* is used in RW, and the optimal *β* is case‐dependent, since no fixed perturbation pattern is observed in all the three cases. In contrast, the segmentations are stable relative to different *β* used in SPARSE for the synthetic phantom and all the three clinical cases, implying that the segmentation is essentially independent of the selection of *β*. Similar results are also obtained in other evaluated cases, though only three outputs are depicted in Fig. 7 for the clarity of comparison. Therefore, we empirically set β=90 for SPARSE, and the optimal ones are used for RW for all the other comparison studies in this study.

### D. Influence of parameter P_T_

**Figure 8:**
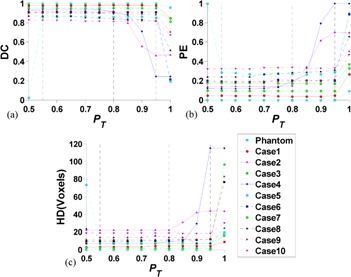
The DC (a), PE (b), and HD (c) by SPARSE with different $P_{T}$ for the synthetic phantom and cases 1‐10. The solid (phantom and cases 1‐4) and dashed (case 5‐10) lines correspond to the 2D and 3D cases, respectively.

Figure 8 presents the segmentation results response to different $P_{T}$ in the synthetic phantom and ten segmentation cases (cases 1‐10). We have the following two observations. First, the performance of SPARSE is stable when small $P_{T}$ is used, and degenerates as $P_{T}$ increases to a certain degree in all evaluated cases. The turning points are around 0.8 and 0.95 for 2D and 3D, respectively. Theoretically, larger $P_{T}$ implies stricter growing condition; in other words, fewer points will be included into the ESPs chain. If no new points are grown into ESPs, the SPARSE will degenerate to RW. Secondly, when small $P_{T}$ is used (*P_T_* ≤ 0.5), the ESPs might be overgrown, or one point might be assigned to more than one label. Case 5 in Fig. 8 is such a typical case that failed segmentation is obtained when $P_{T}=0.5$ is used. Based on these observations from both the phantom and clinical cases, we hence heuristically assume that an appropriate range for $P_{T}$ is [0.6, 0.9] and we empirically set $P_{T}=0.8$ for all the evaluated cases in this study.

### E. More clinical cases

**Figure 9:**
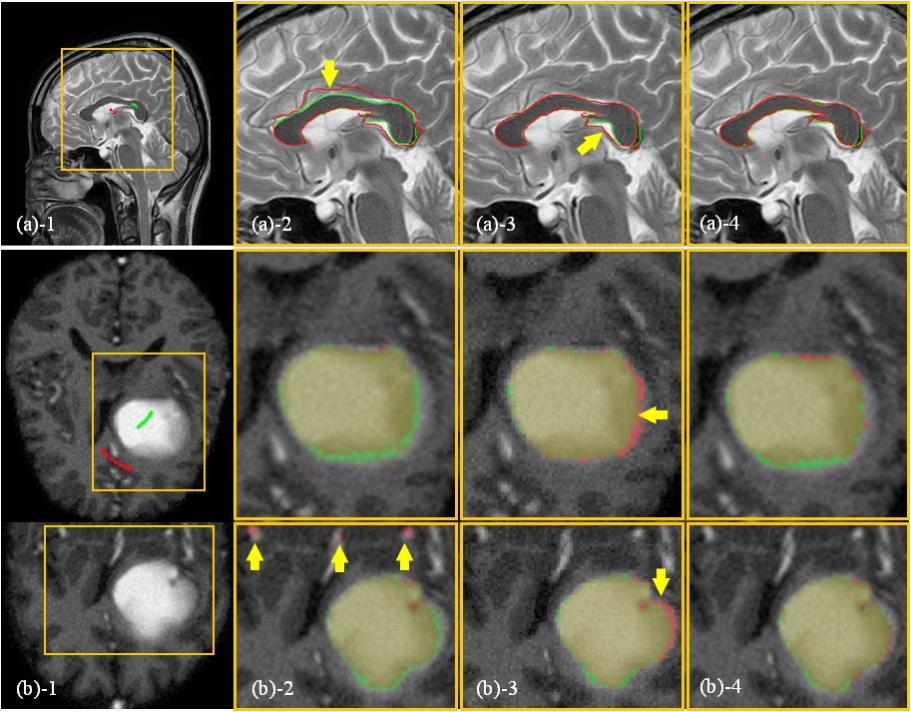
Segmentation of homogenous targets: (a) the corpus callosum in a T2 MR image (case 3) and (b) the glioma in a T1c MR image (case 7). The ROIs, which are indicated by the yellow boxes, are shown in the zoomed‐in views for clarity. (a)−1 SPs labeled inside (green) and outside (red) of the corpus callosum; (a)−2 segmentation by RW; (a)−3 segmentation by GC; (a)−4 segmentation by SPARSE. The red and green curves in (a)−2,−3,−4 represent the calculated and ground truth segmentations, respectively. The yellow arrow in (a)−2,−3 indicates the oversegmentation; (b) segmentation of a glioma in a T1c MR image (case 7): (b)−1 SPs labeled inside (green) and outside (red) of the brain glioma on only one transversal slice; (b)−2 segmentation by RW; (b)−3 segmentation by GC; (b)−4 segmentation by SPARSE. Upper and lower rows in (b) are the transversal and coronal slices, respectively. The red and green masks in (b)−2,−3,−4 represent the calculated and ground truth segmentation; the yellow arrows in (b)−2,−3 indicate the oversegmentation.

Figure 9 illustrates the comparison of results of segmenting relatively homogeneous targets. Figure 9(a) shows the segmentation comparisons of the corpus callosum in a T2 MR image (case 3). Since only limited SPs are labeled in this case, oversegmentation is observed in RW and GC (arrow in Fig. 9(a) −2,−3), while the SPARSE yields a much better segmentation result (Fig. 9(a) −4). Figure 9(b) shows a case of segmenting a glioma in a T1c MR image (case 7). We can see that oversegmentations are sparsely distributed in some voxels outside of the target region in RW (arrows in Fig. 9(b) −2), and slight oversegmentation is also observed in GC (arrows in Fig. 9(b) −3). Comparatively, the SPARSE can generate more accurate results (Fig. 9(b) −4).

**Figure 10:**
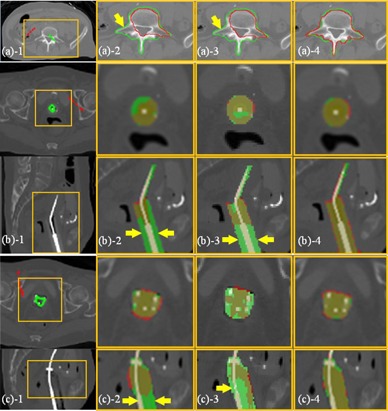
Segmentation of inhomogeneous targets (CT). The ROIs, which are indicated by the yellow boxes, are shown in the zoomed‐in views for clarity. (a) The vertebra in CT image (case 4): (a)−1 SPs labeled inside (green) and outside (red) of the vertebra; (a)−2 segmentation (red curves) by RW; (a)−3 segmentation (red curves) by GC; (a)−4 segmentation (red curves) by SPARSE. The green curves in (a)−2,−3,−4 represent the ground truth segmentation, and the yellow arrow in (a)−2,−3 indicates undersegmentation in RW; (b) and (c), the T&C applicator and the T&O applicator in CT image respectively (case 5, 6): (b‐c)−1 SPs labeled inside (green) and outside (red) of the applicator on only one transversal slice; (b‐c)−2 segmentation (red masks) by RW; (b‐c)−3 segmentation (red masks) by GC. (b‐c)−4 segmentation (red masks) by SPARSE. Rows in (b) and (c) are transversal and sagittal slices, respectively. The green masks in (b)−2,−3,−4 and (c)−2,−3,−4 represent the ground truth segmentation. The yellow arrows in (b‐c)−2,−3 indicate the undersegmentation in RW and GC, respectively.

Figure 10 illustrates the comparison results of segmenting inhomogeneous targets in CT images. Figure 10(a) is the segmentation of the vertebra (case 4). Undersegmentation is observed in RW and GC (arrow in Fig. 10(a) −2,−3), while SPARSE can yield satisfactory result (Fig. 10(a) −4). Figures 10(b) and (c) are the segmentations of two different types of applicator in CT images: T&C applicator and T&O applicator, respectively (cases 5, 6). The RW and GC produce severe undersegmentation (Figs. 10(b) −2 and (c)−2, (b)−3 and (c)−3), while the SPARSE can generate accurate result (Figs. 10(b) −4 and (c)−4).

**Figure 11:**
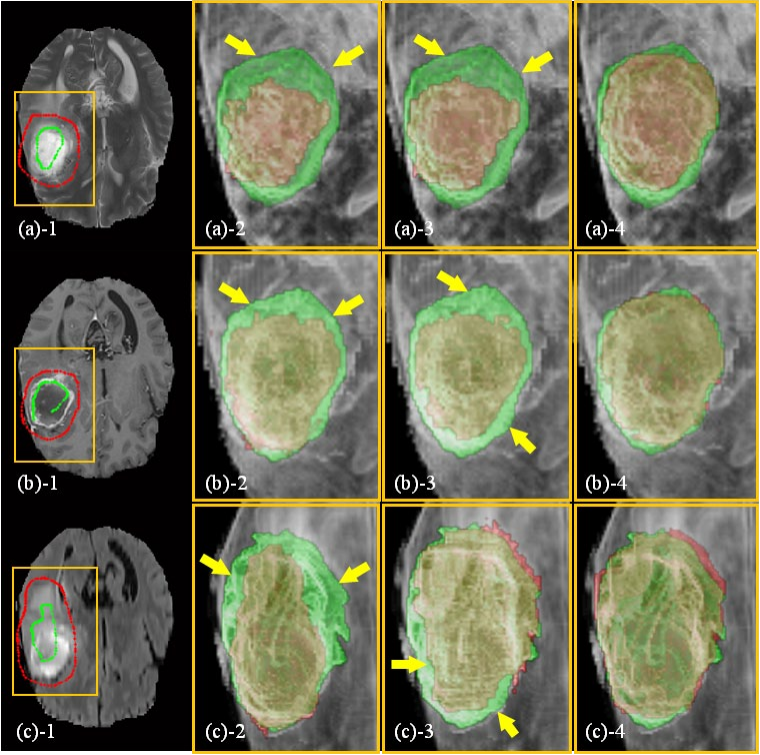
Segmentation of inhomogeneous targets (MR): (a) the glioma in T2 MR image (case 8), (b) T1c MR image (case 9), and (c) the glioma and edema in FLAIR MR image (case 10). The ROIs, which are indicated by the yellow boxes, are shown in the zoomed‐in views for clarity. First column: SPs labeled inside (green) and outside (red) of the targets on one transversal slice of T2 ((a)−1), T1c ((b)−1), and FLAIR ((c)−1) MR image. Second column: segmentation by RW. Third column: segmentation by GC. Fourth column: segmentation by SPARSE. In the second, third, and fourth columns, the red and green masks represent the calculated and the ground truth segmentations, respectively, and the images are rendered in 3D.

Figure 11 shows another inhomogeneous case of segmenting a glioma in T2, T1c MR images (cases 8, 9) (Fig. 11(a) and (b)), and glioma and edema in FLAIR MR image (case 10) (Fig. 11(c)), respectively. Undersegmentation are also observed in RW (Figs. 11(a) −2 and (c)−2) and GC (Figs. 11(a) −3 and (c)−3); in contrast, SPARSE can generate more accurate results (Figs. 11(a) −4 to (c)−4).

### F. Quantitative evaluations

The quantitative comparison results for all the 3D cases (cases 5‐24) are listed in Table 2. We can see that SPARSE is superior to RW and GC in terms of segmentation accuracy for all the evaluated cases in all the three metrics. For RW, the mean DC, PE, HD, and their corresponding standard deviation are 0.47±0.19, 0.69±0.18, and 50.74±31.17, respectively. For GC, the mean DC, PE, HD are 0.59±0.17, 0.58±0.17, and 15.62±5.97, respectively, which are slightly better than RW. For SPARSE, in contrast, the DC increase to 0.89±0.03, the PE and HD decrease to 0.23±0.07, and 8.24±3.95, respectively.

**Table 2:** Comparisons of DC, PE, and HD between RW, GC, and SPARSE of 3D cases

*3D Cases*		*DC*			*PE*			*HD (voxels)*		
*Case #*	*Image Type*	*RW*	*GC*	*SPARSE*	*RW*	*GC*	*SPARSE*	*RW*	*GC*	*SPARSE*
7, 17, 18	T1c MR(−)	0.61 (±0.31)	0.67 (±0.18)	0.89 (±0.05)	0.54 (±0.35)	0.48 (±0.23)	0.22 (±0.11)	42.78 (±46.37)	11.38 (±6.80)	6.31 (±2.87)
5, 11, 12	HDR CT with T&C applicator(+)	0.44 (±0.13)	0.49 (±0.15)	0.89 (±0.05)	0.72 (±0.11)	0.67 (±0.12)	0.21 (±0.01)	70.72 (±50.26)	10.10 (±3.91)	6.90 (±2.09)
6, 13‐16	HDR CT with T&O applicator(+)	0.37 (±0.17)	0.47 (±0.20)	0.90 (±0.01)	0.79 (±0.13)	0.68 (±0.21)	0.23 (±0.05)	63.45 (±14.19)	17.68 (±5.91)	4.85 (±2.21)
8, 19, 22	T2 MR(+)	0.40 (±0.13)	0.70 (±0.10)	0.92 (±0.04)	0.75 (±0.10)	0.53 (±0.11)	0.17 (±0.07)	40.83 (±30.14)	16.05 (±2.85)	8.70 (±2.06)
9, 20, 23	T1c MR(+)	0.54 (±0.13)	0.57 (±0.14)	0.88 (±0.01)	0.63 (±0.11)	0.60 (±0.14)	0.26 (±0.03)	35.56 (±27.83)	16.70 (±6.84)	12.66 (±4.85)
10, 21, 24	FLAIR MR(+)	0.50 (±0.26)	0.50 (±0.26)	0.86 (±0.02)	0.65 (±0.20)	0.65 (±0.20)	0.28 (±0.05)	37.86 (±5.72)	20.42 (±3.82)	12.37 (±2.30)
Mean		0.47 (±0.19)	0.59 (±0.17)	0.89 (±0.03)	0.69 (±0.18)	0.58 (±0.17)	0.23 (±0.07)	50.74 (±31.17)	15.62 (±5.97)	8.24 (±3.95)

−=homogeneous; +=inhomogeneous

### G. Computational efficiency

All the experiments in this study were conducted on a GPU platform with an NVIDIA Telsa C1060 card with a total number of 240 processors of 1.3 GHz. It is also equipped with three GB DDR3 memory, shared by all processors. The mean computation time is 0.44±0.10 s for four 2D cases (cases 1‐4) and 38.63±9.41 s for twenty 3D cases (cases 5‐24), respectively. Table 3 lists all computational time for all the 3D cases. It can be seen that the computational time depends on the complexity of the cases tested.

**Table 3:** Computational times for all the 3D cases

*Image Size (case #)*	256×256×55 *(6)*	256×256×58 *(15)*	256×256×60 *(12,14)*	256×256×75 *(13)*	256×256×82 *(16)*	256×256×85 *(5,11)*	256×256×100 *(7,17,18)*	160×216×176 *(8‐10,19‐24)*
Time (s)	22.33	23.17	24.55±0.02	31.20	32.85	39.18±7.32	40.46±0.17	46.02±4.13

## IV. DISCUSSION & CONCLUSIONS

In this paper, we presented a RW‐based segmentation algorithm, SPARSE, which incorporates a novel SPs autogeneration scheme for segmentation of inhomogeneous targets. Evaluations of segmentation tasks in the clinical images reveal that the SPARSE decreases the sensitivity to the initial seed points in terms of location and quantity, as well as the dependency of the free parameter *β* in edge weighting function. With GPU implementation, the robustness and accuracy of the proposed method has been demonstrated with various tested cases in the segmentation of inhomogeneous objects, especially for 3D cases.

One merit of the proposed SPs autogeneration in SPARSE is that the influence of the initial SPs placement to the ultimate segmentation can be reduced. The sensitivities of both SPs quantity and location are evaluated by visual inspection, as well as quantitative analysis. It has been shown that variation of segmentation performance in SPARSE is negligible when the number of SPs is reduced or the locations of SPs are changed randomly, and SPARSE generally produces superior segmentation results than RW when SPs are perturbed. Another gain of the SPs expansion strategy is the reduced workload and labor in manual SPs selection, making the SPARSE algorithm a practical tool for segmentation tasks in clinics. Manual SPs labeling is tedious and usually time‐consuming for most of the interactive segmentation algorithms. In SPARSE, however, the user only needs to specify several SPs on one or a few slices on a 3D volume instead of carefully seeding all through the slices to obtain a satisfactory segmentation. This will significantly facilitate the clinical workflow.

In practice, more SPs usually imply more target/background intensity information, which can theoretically maximize the performance of RW. SPARSE can facilitate collecting such intensity information to a feasible extent with only limited user interaction, and it is this fact that more robust segmentation can be expected given the “incremented” information by incrementing the SPs. Although less initial SPs dependence is achieved in SPARSE, to guarantee appropriate SPs autogeneration, the initial SPs still need to be labeled on locations that are representative of different intensity levels in the target/background instead of seeding arbitrarily. Furthermore, SPs autogeneration is in favor of the *β* selection. Choosing an appropriate *β* for RW is a nontrivial task since *β* is essentially case dependent, and the segmentation performance can vary dramatically when different *β* is used. However, the SPARSE can weaken this dependence with incremental SPs, which has been demonstrated in this study.

In (6), (7), we set the tuning parameter $\sigma =0.5\times D_{I}$, where $D_{I}$ is the mean difference of the intensity between the labeled SPs inside and outside the target, to keep the choice of σ relevant to the intensity contrast of the target and the background. This approach is shown to be effective in distinguishing the low contrast targets.

In SPARSE, the threshold $P_{T}$ controlling the SPs growth is empirically chosen based on experiments of ten segmentation tasks. Larger $P_{T}$ usually implies stricter growing condition. However, one should note that $P_{T}$ is not the only factor contributing to the SPs growth. Connectivity of voxels in the probability map is another factor, which is purely relative to the textural characteristics of the image. Therefore, potential overgrowth of SPs is theoretically possible even if small *PT* is used, in which case, manual intervention (e.g., deleting certain undesired incremented SPs) is then necessary. According to our observation, satisfactory segmentation performances can be obtained when $P_{T}$ ranges in [0.6, 0.9]. We have used a relatively rigorous $P_{T}=0.8$ to filter out those points with high similarity to the user‐labeled SPs and include them into the SPs chain. For simplicity, we heuristically use the same $P_{T}$ for each label *s,* though this value might not be optimal for all labels. More sophisticated methods (e.g., adaptive approaches) need to be investigated for a better selection of $P_{T}$, and we would like to include this work into our future study.

## ACKNOWLEDGMENTS

This work is supported in part by the National Natural Science Foundation of China (No. 81301940 and No. 81428019). Brain tumor image data used in this work were obtained from the MICCAI 2012 Challenge on Multimodal Brain Tumor Segmentation (http://www.imm.dtu.dk/projects/BRATS2012) organized by B. Menze, A. Jakab, S. Bauer, M. Reyes, M. Prastawa, and K. Van Leemput. The challenge database contains fully anonymized images from the following institutions: ETH Zurich, University of Bern, University of Debrecen, and University of Utah.
